# Editorial: Nutritional indicators and implications for human health, volume II

**DOI:** 10.3389/fnut.2026.1931290

**Published:** 2026-07-21

**Authors:** Virginia Maria Muniz, Abraham Wall-Medrano, Fabiano Kenji Haraguchi, Luciane Bresciani Salaroli

**Affiliations:** 1 Graduate Program in Nutrition and Health of the Federal University of Espírito Santo, Vitória, Brazil; 2 Institute of Biomedical Sciences, Autonomous University of Ciudad Juárez, Ciudad Juárez, Mexico

**Keywords:** biomarkers, malnutrition, nutritional assessment, nutritional status, nutritional transition, prognostic nutritional indices

Nutritional assessment has shifted toward an integrative paradigm that synthesizes anthropometric, biochemical, metabolic, and genetic data. By prioritizing individualized biochemical analysis and proactive disease prevention, this model enables targeted clinical interventions rooted in cellular metabolism and nutrient-genome interactions (1).

Traditional nutritional diagnosis relies on the classic model: anthropometric, biochemical, clinical, and dietary data. While effective for establishing baseline population trends, this framework is now outdated. Rapid dietary transitions, aging populations, and the coexistence of undernutrition and obesity have exposed major limitations in this classic approach (2).

Public health nutrition examines dietary quality and disparities across diverse population subgroups. When these disparities stem from unjust, modifiable social and structural factors, such as income and education, they constitute dietary inequities. However, sub-optimal diet quality is a global phenomenon spanning the entire socioeconomic spectrum. Attributing consumption inequities solely to the financial cost of healthy food is insufficient; educational attainment serves as an equally critical structural driver of these disparities (3).

Nutritional support therapy is essential in hospital settings to reduce the severe effects of malnutrition, a condition highly prevalent in critical care. Recent global studies estimate that 78% of critically ill patients enter the hospital at high nutritional risk or become malnourished during their stay due to severe catabolic stress. Because of this rapid metabolic decline, targeted nutritional support is vital for these patients (4).

Bridging clinical precision and public health needs, the Research Topic “*Nutritional indicators and implications for human health, volume II*” compiles 16 studies that provide robust evidence on nutritional indicators, dietary intake, and food quality. Spanning both population and clinical settings, this Research Topic offers a holistic framework that translates integrative nutrition science into clinical practice, public policy formulation, and advanced scientific research.

Several investigators have evaluated the relationship between nutritional support, biomarkers, and inpatient outcomes. In China, Zhao et al. assessed the prognostic value of the Controlling Nutritional Status (CONUT) Score, the Prognostic Nutritional Index (PNI), and the Neutrophil Percentage-to-Albumin Ratio in predicting short-term outcomes for patients with acute ischemic stroke following reperfusion therapy. Addressing critical care protocols, Sun et al. developed a collaborative, multidisciplinary process to optimize nutritional therapy in the intensive care unit. Shifting focus to prognostic biomarkers, Li et al. established a clear correlation between the PNI and all-cause mortality following ischemic stroke. Zhou et al. quantified the exact prevalence of vitamin D deficiency and identified key factors influencing vitamin D status across a large, diverse cohort to pinpoint the most vulnerable patient subgroups. Concurrently, a South Korean study by Liu, Zheng et al. demonstrated that both the C-reactive protein-to-albumin ratio and the CRP–triglyceride–glucose index are significantly linked to 90-day functional outcomes in patients with acute ischemic stroke.

This topic is further enriched by six population-based studies conducted across low- and middle-income countries. Within the African context, Sinkhonde et al. investigated how insufficient intake of protein, lysine, and tryptophan impairs growth among Malawian children. Within the Chinese cohort, Cai et al. associated the sarcopenia index with depressive symptoms, while Liu, Li et al. evaluated the prevalence of hyperuricemia among oil industry workers using variations of the triglyceride-glucose index. Shifting focus to South America, Silva et al. utilized the conicity index to estimate cardiovascular risk across Brazil in relation to sociodemographic determinants, while Fogaça et al. detailed the methodological standardization of 24-h dietary recalls for the PROVEN-DIA study. Concurrently, Seker et al. investigated the impact of lifestyle modifications on macronutrient intake and anthropometric profiles among adults in Turkey.

Five studies targeted populations within high-income countries. In the United Kingdom, Bao and Duan examined the association between metabolic and body mass index phenotypes and the incidence of osteoarthritis among middle-aged and older adults. In the United States, Ma et al. utilized data from the National Health and Nutrition Examination Survey (NHANES) to evaluate the impact of magnesium depletion scores on all-cause, cancer, and cardiovascular disease mortality among survivors of myelodysplastic syndromes. Shifting focus to continental Europe, Christie et al. analyzed the policy implications of incorporating dietary supplements into public health strategies to optimize nutritional status, mitigate chronic disease risk, boost workforce productivity, and reduce healthcare costs. Shifting the focus to consumer behavior, Bawazeer et al. evaluated public perceptions of five front-of-pack labeling systems in Saudi Arabia. Meanwhile, Fernández et al. validated the World Cancer Research Fund/American Institute for Cancer Research screener for young adults in Spain, mapping the interconnections of dietary variables using network analysis.

In summary, the research compiled within this Research Topic offers a robust, multifaceted overview of nutritional indicators, validating their utility in both individualized clinical care and population-level epidemiological mapping. The methodologies and insights presented here extend far beyond academia, serving as indispensable technical-scientific tools to guide clinical decision-making. Furthermore, they provide a structured framework for public administrators to design, monitor, and optimize interventions regarding food security, nutritional safety, and chronic disease prevention.
